# Evaluated School-Based Exercise Interventions with Nutritional Supplementation in Obese Children and Adolescents: A Systematic Review of Randomized Controlled Trials for Highlighting a Research Gap

**DOI:** 10.3390/metabo16070477

**Published:** 2026-07-07

**Authors:** Markel Rico-González, Damiano Formenti, Carlos D. Gómez-Carmona, Luca Paolo Ardigò

**Affiliations:** 1 Department of Didactics of Music, Plastic and Body Expression, University of the Basque Country (EHU), 48940 Leioa, Spain; 2 BioVetMed & SportSci Research Group, University of Murcia, 30100 Murcia, Spain; carlosdavid.gomez@ua.es; 3 Department of Biotechnology and Life Sciences, University of Insubria, 21100 Varese, Italy; damiano.formenti@uninsubria.it; 4 Physical Education and Sport Area, Department of General and Specific Didactics, Faculty of Education, University of Alicante, 03690 San Vicente del Raspeig, Spain; 5 Research Group in Training, Physical Activity and Sports Performance (ENFYRED), Department of Music, Plastic and Body Expression, University of Zaragoza, 44003 Teruel, Spain; 6 Department of Teacher Education, NLA University College, 0166 Oslo, Norway

**Keywords:** nutrients, supplementation, education, physical activity, systematic review, randomized controlled trials, vitamin, health

## Abstract

**Background/Objectives**: The systematic review aims to highlight the effects of school-based physical activity and nutritional supplementation programs in obese children from preschool to high school, based on randomized controlled trials. **Methods**: The search strategy was designed based on the PICOS framework. Then, a systematic review of relevant articles was conducted across six databases (Cochrane Library, PubMed, ProQuest, SCOPUS, Web of Sciences and SPORTDiscus) to identify articles that included children from preschool to high school who engaged in physical activity at school and received nutritional supplementation. All the included studies were assessed using the RoB-2 checklist. **Results**: Of the 234 studies reviewed, six met the inclusion criteria for this systematic review (publication years: 2023–2025). The results revealed highly heterogeneous interventions and mixed outcomes, often influenced by factors such as supplement type, dosage and participant gender. This limited and inconsistent body of evidence underscores a significant gap in the literature concerning the combined effects of school-based exercise and nutritional supplementation in obese youth. **Conclusions**: Limited evidence suggests mixed results with multi-micronutrient supplementation showing some benefits in boys from resource-limited settings, while several interventions demonstrated no effects. Energy-dense supplementation proved counterproductive. Gender-stratified approaches are recommended, but cautious implementation is warranted given methodological limitations and inconsistent findings.

## 1. Introduction

Childhood obesity represents one of the most urgent public health problems [1,2]. Recent estimates indicate that millions of children and adolescents worldwide are affected by overweight and obesity, with prevalence increasing across both developed and developing regions [3]. In fact, the World Health Organization (WHO) estimated that in 2022, approximately 390 million children and adolescents aged 5–19 years were overweight or obese, with the prevalence more than doubling from 8% in 1990 to 20% in 2022 [4]. Excess body fat during childhood not only predisposes individuals to insulin resistance, metabolic syndrome and cardiovascular diseases, but also increases the risk of psychological distress and the persistence of obesity into adulthood [5]. Therefore, addressing this multifactorial condition early in life is of paramount importance and constitutes a health priority [6].

Regular physical activity (PA) is widely recognized as a key determinant of metabolic and cardiovascular health in youth. Exercise-based interventions, particularly those that include both aerobic and resistance components, have demonstrated significant benefits for improving body composition, insulin sensitivity, and systemic metabolic function in youth with overweight or obesity [5,7,8]. Importantly, programs combining aerobic and. Resistance exercise appears to confer the greatest benefits [8]. However, while PA alone is effective, interventions that integrate both exercise and dietary components consistently produce greater and more sustainable improvements in body weight and cardiometabolic health than diet-only approaches [9,10].

Given their structured environment and potential for large-scale implementation, schools are considered an optimal setting for health promotion. School-based programs include consistent exposure to PA (for example, through physical education classes), nutrition education and behavioral support, fostering sustainable and healthy lifestyles in children and adolescents [3]. In recent years, growing attention has been devoted to the role of nutritional supplementation as an addition to school-based exercise programs. Multi-micronutrient fortification and similar strategies may enhance the physiological adaptations to training, improving growth and metabolic profiles, particularly in contexts where micronutrient deficiencies coexist with overweight or obesity [9,11]. This concept, defined as a “double-duty” approach, aims to address both undernutrition and overnutrition simultaneously, which represent increasingly relevant goals in settings undergoing rapid nutritional and lifestyle transitions [11].

Although the potential of this type of intervention is promising, the available evidence on the combined effects of school-based exercise programs and nutritional supplementation in obese youth remains limited and inconclusive. A wide variety of outcomes was reported in the literature, depending on supplement composition, dosage, intervention duration, and participant characteristics, such as age, sex, and baseline nutritional status. Moreover, sex-related differences in growth and hormonal responses during adolescence may influence how individuals adapt to exercise and supplementation, suggesting that gender-specific approaches may be a viable strategy [7,8].

Numerous systematic reviews have evaluated PA or nutritional interventions independently [3,5,7,8,9,10,11]. However, only a few of them have specifically examined their combined implementation within school environments targeting obesity. Understanding the interaction between exercise and supplementation is crucial to optimizing school-based programs [12,13,14], improving health outcomes and guiding evidence-based policy in pediatric populations.

Accordingly, the present study aimed to synthesize and evaluate randomized controlled trials investigating the effects of school-based physical activity interventions combined with nutritional supplementation in children and adolescents with overweight or obesity.

## 2. Materials and Methods

### 2.1. Experimental Approach to the Problem

A systematic review was performed in accordance with PRISMA (Preferred Reporting Items for Systematic Reviews and Meta-Analyses) guidelines [15] and guidelines for performing systematic reviews in sport sciences [16]. The systematic review was registered on PROSPERO **(CRD420261432412)**.

### 2.2. Information Sources

A systematic search of six main databases (Cochrane Library, PubMed, ProQuest, SCOPUS, Web of Sciences and SPORTDiscus) was performed to identify articles published before 9 July 2025.

### 2.3. Search Strategy

The PICO (Patient, Problem or Population—Intervention or Exposure—Comparison, Control or Comparator—Outcome[s]) design was used to provide an explicit statement of the question. Where possible, the search was limited to scientific articles and journals in the specified language (see exclusion criterion 6). The author was not blinded to journal names or manuscript authors. The search strategy was applied in the databases listed above. All articles were downloaded and analyzed for eligibility using the inclusion-exclusion criteria one by one. If an article met all inclusion criteria, then it was downloaded, included in the review and its data were extracted and presented in the main table of the results section. If an article did not meet all the inclusion criteria, then it was deleted, and the reason was detailed. The following search terms were used in articles’ titles and abstracts (see Table 1):

(preschool* OR kindergarten OR school OR “elementary education” OR “primary education” OR high-school OR “secondary education” OR “secondary school” OR adolescent*) AND (obese OR overweight) AND (supplement* OR vitamin OR calcium OR micronutrient* OR omega OR zinc OR iron) AND (intervention OR program*) AND (exercise OR “Physical activity” OR “physical education” OR sport OR fitness OR aerobic) AND (“randomized controlled trial”)

### 2.4. Eligibility Criteria

To identify information from the articles, one author downloaded the information (titles, authors, dates and databases) and transferred it into an Excel spreadsheet (Microsoft Corporation, Redmond, WA, USA), where duplicates were removed. Two authors screened the remaining articles to select those articles that met all inclusion criteria (Table 1). Two disagreements were identified, but they were solved without a third author. Moreover, when relevant articles not previously identified were also screened identically and further studies that complied with the inclusion–exclusion criteria were included and labeled as “included from external sources”.

### 2.5. Data Extraction

Data extraction was prepared using an Excel spreadsheet in accordance with the Cochrane Consumers and Communication Review Group’s data extraction template. The spreadsheet was used to assess inclusion and exclusion requirements for all selected studies. Full-text articles that were excluded from the analysis were recorded with reasons for exclusion. All records were stored in the spreadsheet. One author performed the data extraction. However, if any doubt arose, then a second author was consulted.

Once all records were selected and downloaded, the information of each of them was extracted: the characteristics of the study population (e.g., average age, geographic context), detailed descriptions of the nutritional and physical-activity interventions, variables and outcome measures, key results and the authors’ conclusions together with their practical applications.

### 2.6. Quality of Studies

The second version of the Cochrane risk-of-bias tool for randomized trials (RoB 2) was used. The authors assessed different key domains: random sequence generation (item 1), allocation concealment (item 2), blinding of participants and personnel (item 3), blinding of outcome assessment (item 4), incomplete outcome data (item 5), selective reporting (item 6) and other bias (item 7). Each domain was evaluated and classified using low risk of bias (+), high risk of bias (−) or some concerns or unclear risk of bias (?). Two authors conducted RoB 2.0 assessments, with disagreements resolved through discussion or consultation with a third author.

## 3. Results

### 3.1. Identification and Selection of Studies

**Figure 1:**
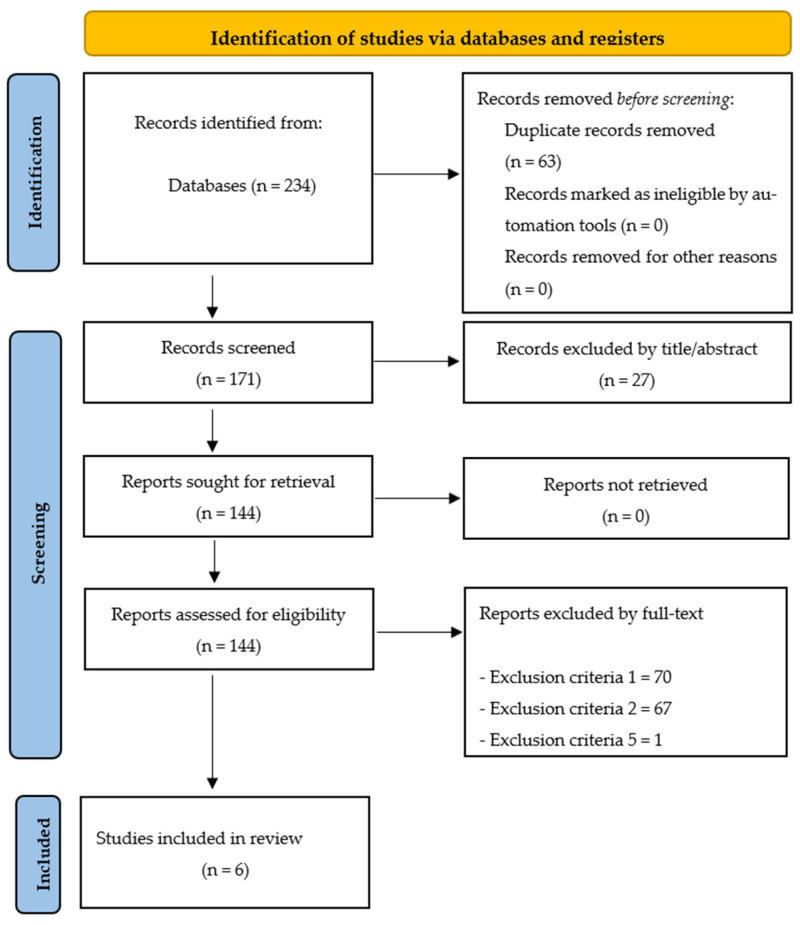
Flow diagram of the study.

A total of 234 original articles were retrieved (PubMed (n = 10), ProQuest (n = 5), SCOPUS (n = 105), Web of Sciences (n = 7), Cochrane Library (n = 105), SPORTDiscus (n = 1) and external sources (n = 1)), from which 63 duplicates were removed, resulting in 171 unique records. Following title and abstract screening, 27 articles were excluded for not meeting inclusion criterion six. The full text of the remaining 144 articles was reviewed, leading to the exclusion of 70, 67 and 1 articles based on exclusion criteria 1, 2 and 5, respectively. Consequently, 6 articles fulfilled all inclusion criteria and were included in the final qualitative synthesis (see Figure 1).

### 3.2. Quality Assessment

The quality assessment for this systematic review is presented in Table 2.

### 3.3. Characteristics of Included Studies

Of the six studies reviewed, all were randomized controlled trials examining school-based physical activity interventions in combination with nutritional supplementation for children and adolescents living with overweight or obesity. The trials, published between 2013 and 2025, took place across a wide range of settings, including the United States, Tanzania, South Africa and Iran, reflecting diverse geographical regions and socioeconomic conditions.

Sample sizes were highly variable, from as few as 90 participants to more than 1300, and the age groups covered spanned from early childhood (6 years) to mid-adolescence (16 years). The nutritional strategies also differed considerably: some trials used fluid milk, others used multi-micronutrient supplements (MMNS), and still others used energy-dense ready-to-use supplementary food (RUSF). The exercise components were equally diverse, ranging from supervised resistance training and structured PE classes to dance sessions, classroom activity breaks and even high-intensity interval training (HIIT). Intervention lengths varied from 10 weeks to just over two years, though several programs were cut short or disrupted by the COVID-19 pandemic.

The overall picture was mixed. Some evidence pointed to gender-specific effects; for example, MMNS appeared to support lean mass gains in boys, while other interventions, such as milk supplementation alongside exercise, showed little or no added benefit beyond physical activity alone. These inconsistencies highlight how sensitive outcomes can be to factors such as the type and dosage of the supplement, the timing of intake, baseline nutritional status and participant characteristics. In short, the findings underline the difficulty of designing one-size-fits-all programs (see Table 3).

## 4. Discussion

Childhood obesity represents a critical global health challenge, and combined diet-plus-exercise interventions are superior to diet-only approaches for weight management [10]. School-based interventions demonstrate particular promise due to their potential for wide reach and sustained impact [3]. This systematic review aimed to evaluate the effects of school-based exercise interventions with nutritional supplementation in obese children and adolescents through randomized controlled trials. Our findings reveal a significant research gap: only 6 studies met the inclusion criteria, demonstrating highly heterogeneous interventions and mixed outcomes, often influenced by supplement type, dosage, and participant gender.

The heterogeneity of interventions and outcomes observed across studies reflects the complexity of implementing combined programs in educational settings, with results often presenting a mixed picture, influenced by supplement type, dosage, and population characteristics. Some studies utilizing MMNS in resource-limited settings showed benefits with significant gender-specific improvements in body composition [12,14,18], while Nqweniso et al. [12] demonstrated that energy-dense supplementation (RUSF) showed no beneficial effect and may have contributed to weight gain. These findings align with recent network meta-analyses demonstrating that physical activity interventions are most effective for reducing BMI z-scores, and that multi-component approaches are superior for BMI reduction [19,20]. The observed benefits of MMNS on lean mass accumulation are particularly significant in interventions that simultaneously address undernutrition and overweight [21,22].

In contrast, fluid milk supplementation alongside resistance training showed no additional benefits compared to isocaloric alternatives [17], which contrasts with systematic reviews suggesting that protein supplementation has positive effects on body composition in youth [23]. This inconsistency is likely explained by a combination of an insufficient protein dosage relative to body mass, a training frequency of 3 sessions per week, and the moderating effect of baseline adiposity, which can attenuate anabolic responses through hormonal and metabolic mechanisms [23]. These observations highlight that supplementation effects are highly context-dependent, and that future trials should standardize the protein dose relative to body mass, adjust training frequency, and stratify analyses by baseline adiposity to clarify under which conditions supplementation provides meaningful additional benefit.

The gender-specific responses observed across some studies represent a noteworthy finding with important implications for intervention design. In studies where effects were observed, boys showed greater responsiveness to MMNS interventions, with significant increases in fat-free mass. In contrast, girls exhibited different patterns, including reductions in both fat and lean mass with physical activity interventions [12,14]. However, these patterns were not universal across all studies; Lambourne et al. [17] reported different trends. These results align with recent evidence indicating sex-related differences in exercise responses during adolescence, likely reflecting hormonal influences on muscle protein synthesis and energy metabolism [24]. Recent Cochrane updates suggest that interventions may be particularly effective in boys aged 5–11 years compared with girls, highlighting the need for gender-stratified program design [25]. The differential responses are consistent with observations from comprehensive school-based intervention reviews, which demonstrate varying effectiveness patterns by gender across intervention components [26].

The effectiveness of interventions appeared strongly influenced by population characteristics and contextual factors. Studies conducted in low- to middle-income countries generally showed more pronounced effects than those in high-income settings [17], consistent with systematic reviews indicating that school-based interventions demonstrate larger effect sizes in lower-middle- to upper-middle-income countries than in high-income countries [27]. This pattern likely reflects a higher baseline prevalence of micronutrient deficiencies and greater potential for improvement in resource-limited settings [28]. However, the finding that energy-dense supplementation (RUSF) may have contributed to weight gain rather than improvement [13] provides important cautionary evidence against inappropriate targeting, consistent with concerns about nutrition transitions in developing countries [29]. Additionally, Doaei et al. found no significant correlations between changes in vitamin D levels and anthropometric measurements, suggesting that vitamin D status may be a marker of obesity rather than a modifiable target for short-term interventions [18].

The high heterogeneity observed across the six included trials can be attributed to three main sources: supplement type, socioeconomic context and intervention duration. Regarding supplement type, studies using MMNS in resource-limited settings consistently reported benefits in lean mass accumulation, particularly in boys [4,13,14]. In contrast, fluid milk supplementation alongside resistance training showed no additional benefit compared to isocaloric alternatives [17], and energy-dense RUSF supplementation was associated with potential weight gain in already overnourished populations [12]. These contrasting outcomes suggest that supplement type must be matched to the baseline nutritional profile of the target population, as the same supplementation strategy may produce opposite effects depending on whether the primary nutritional concern is deficiency or excess. Regarding socioeconomic context, studies conducted in low- and middle-income countries generally reported more pronounced effects than those in high-income settings [12,13,14,18], likely reflecting greater baseline micronutrient deficiencies and a higher potential for improvement [27,28]. Regarding intervention duration, programs ranged from 10 weeks to 26 months, and several were disrupted by COVID-19 school closures [4,14], making it difficult to isolate the effect of duration from implementation fidelity [30,31]. Taken together, these sources of heterogeneity highlight that inconsistent outcomes across trials are not random but rather reflect meaningful differences in design and context, which must be considered when interpreting the evidence and designing future interventions.

The limited duration and follow-up of most interventions (ranging from 10 weeks to 26 months) constrain our understanding of long-term effectiveness. Recent reviews emphasize that longer, multi-setting, comprehensive school-based obesity interventions involving PA, diet, and health education are more effective than either PA or diet interventions alone [29,32]. However, a recent meta-analysis found no difference in efficiency between studies lasting less than 6 months and those lasting more, suggesting that intervention quality and comprehensiveness may be more important than duration alone [31]. A critical limitation identified across multiple studies was the disruption caused by COVID-19 school closures, which significantly affected intervention and follow-up assessments [4,14]. The disruption of several studies by COVID-19 school closures highlights the vulnerability of school-based programs to external factors and the need for adaptive implementation strategies [30]. These observations are consistent with systematic reviews emphasizing the importance of intervention sustainability and comprehensive multi-component approaches that focus on curriculum design and teacher training to ensure long-term effectiveness [18,31].

Recent comprehensive network meta-analyses provide additional context for our findings, demonstrating that physical activity alone ranks highest for reducing BMI z-scores. In contrast, combinations of physical activity with health education and school policy changes show superior effectiveness for overall BMI reduction [19,20]. The growing body of evidence from middle-income countries specifically supports the potential effectiveness of school-based interventions in contexts similar to those represented in our included studies, with particular promise for addressing the dual burden of malnutrition through integrated approaches [27,28]. However, the complexity of implementing such interventions is underscored by recent implementation science research, which highlights the need for a comprehensive assessment of social determinants of health and contextual factors in program design [33].

Several limitations constrain the interpretation of our findings and highlight important gaps for future research. The small number of eligible studies (n = 6) prevents robust evidence synthesis and meta-analysis, reflecting a significant research gap compared to the extensive literature on school-based interventions generally [19,30]. The significant methodological concerns identified through quality assessment, including a high risk of bias across several domains in multiple studies, further limit the strength of the conclusions that can be drawn. The heterogeneity in populations, interventions, and outcome measures, combined with concerns about blinding and incomplete outcome data, limits the strength of the conclusions. The mixed results, with several studies showing null or potentially adverse effects, underscore the need to consider intervention design and target populations carefully. A sensitivity analysis excluding the two trials disrupted by COVID-19 school closures [4,14] was conducted to assess the robustness of the core findings. The remaining four studies [12,13,17,18] maintained consistent patterns, with MMNS continuing to show benefits for lean mass accumulation in boys and physical activity showing effects on fat mass reduction, suggesting that the interrupted trials do not substantially drive the main conclusions. Nevertheless, the reduced sample of studies further underscores the need for well-designed RCTs with complete follow-up to consolidate the evidence base in this field.

Future research should prioritize mechanistic studies incorporating detailed biomarker assessment, including insulin sensitivity, vitamin D status, and zinc levels as pre-specified outcomes, to understand the pathways mediating gender-specific responses, particularly the roles of hormonal factors and micronutrient–metabolic interactions. Key testable research questions include whether gender-stratified MMNS dosing calibrated to pubertal stage and baseline micronutrient status produces superior body composition outcomes compared to uniform supplementation protocols and what minimum intervention duration is needed to sustain reductions in fat mass at 12-month follow-up. Longer-term follow-up studies with standardized outcome measures are essential to assess the sustainability of effects and establish optimal intervention duration, particularly examining whether combined PA plus MMNS reduces fat mass while preserving fat-free mass more effectively than PA alone in low- and middle-income settings, with sex-stratified analyses included as a pre-specified outcome. Implementation science research examining cost-effectiveness, scalability and integration with existing school health programs is crucial for translating findings into effective public health interventions. Finally, investigation of dose–response relationships and development of predictive models based on individual characteristics, including socioeconomic factors and baseline nutritional status, could inform personalized intervention strategies and improve overall effectiveness while addressing health inequities.

## 5. Conclusions and Practical Applications

This systematic review examined school-based interventions that combine physical activity with nutritional supplementation to manage childhood obesity. We identified limited evidence from six randomized controlled trials with significant methodological limitations and highly heterogeneous interventions. Results were mixed and influenced by supplement type, participant gender and socioeconomic context. Multi-micronutrient supplementation showed benefits in some studies, particularly for muscle mass development in boys from resource-limited settings. However, several interventions did not find significant effects, and energy-dense supplementation could be counterproductive. Gender-specific responses were also observed. Boys showed greater responsiveness to micronutrient supplementation, whereas girls exhibited distinct body composition patterns in response to physical activity interventions.

Due to the mixed findings and limited evidence, cautious implementation is warranted. Programs should adopt gender-stratified approaches to micronutrient supplementation based on baseline nutritional status and population characteristics. In resource-limited settings with prevalent micronutrient deficiencies, combined micronutrient supplementation and physical activity may better address the dual problem of malnutrition. Energy-dense supplementation should be avoided unless specifically indicated to prevent weight gain. Implementation should prioritize longer interventions, comprehensive teacher training, and adaptive programs that are not affected by external disruptions.

However, translating these recommendations into practice faces several real-world barriers that must be acknowledged. Supplement procurement costs may limit scalability in low-resource settings, and funding mechanisms should be integrated into existing school nutrition programs to reduce financial burden. Teacher training represents a significant logistical challenge, as sustained implementation requires ongoing professional development and dedicated time within school schedules. School management workload should not be underestimated, as coordinating physical activity sessions, supplementation protocols, and monitoring demands administrative capacity that many schools lack. From a public health policy perspective, cost-effectiveness analyses are urgently needed to determine the feasibility of large-scale promotion, and future programs should be designed with scalability and integration into national school health frameworks as primary objectives.

## Figures and Tables

**Table 1:** Inclusion and Exclusion Criteria for Study Selection.

No.	Item	Inclusion Criteria	Exclusion Criteria	Search Coherence
1	Population	A sample that includes overweight/obese children recruited from kindergartens, elementary schools or secondary schools	No overweight/obese children in the sample. Children not recruited from preschools, primary schools or secondary education centers. Children with medical attention by an illness diagnostic (treatment for cancer). Sport/athlete children not recruited from school (recruited from sport teams, extracurricular activities).	(preschool* OR kindergarten OR school OR “elementary education” OR “primary education” OR high-school OR “secondary education” OR “secondary school” OR adolescent*) AND (obese OR overweight)
2	Intervention/Exposure	Children participating in physical activity program during school hours with nutritional supplementation	Children not participating in physical activity (videogames, virtual reality). Children not receiving supplementation, in addition to physical activity program Intervention with parents as targets. Children receiving supplements in order to address a certain illness. Programs with nutrition suggestion, but not supplementation. Supplementation affecting physical activity levels.	(supplement* OR vitamin OR calcium OR micronutrient* OR omega OR zinc OR iron) AND (intervention OR program*) AND (exercise OR “Physical activity” OR “physical education” OR sport OR fitness OR aerobic)
3	Comparison	-	-	-
4	Outcome(s)	Any	-	-
5	Study Design	Randomized controlled trials	Non-randomized controlled trials	“randomized controlled trial”
6	Other Criteria	Peer-reviewed, original, full-text studies	Non-peer-reviewed, non-original (systematic reviews, meta-analysis) or conference papers	-

**Table 2:** Risk of bias using RoB-2.

Study		Random Sequence Generation	Allocation Concealment	Blinding of Participants and Personnel	Blinding of Outcome Assessment	Incomplete Outcome Data	Selective Reporting	Other Bias
Lambourne et al. [17]		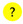	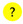	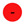	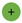	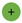	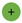	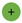
Minja et al. [4]		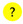	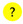	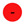	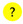	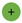	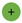	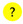
Nqweniso et al. [12]		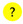	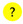	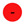	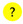	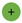	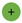	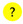
Long et al. [13]		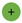	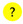	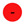	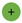	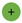	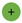	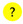
Long et al. [14]		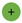	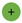	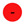	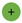	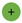	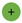	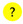
Doaei et al. [18]		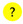	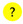	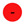	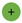	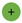	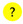	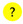
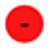	High risk							
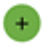	Low risk							
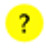	Some concerns							

**Table 3:** Characteristics of included studies.

Authors	Sample Characteristics	Supplement	In-School Physical Exercise Intervention	Variables	Main Results	Conclusions and Applications
Lambourne et al. [17]	Sample: 108 adolescents (39 boys, 69 girls). Age: Mean 13.6 years. Context: Middle-school students from Kansas City, KS, USA. Predominantly overweight (mean BMI percentile = 85th).	Substance: Fluid milk (fat-free chocolate and low-fat white milk). Dosage: 24 oz/day (16 oz post-exercise, 8 oz at lunch on RT days; varied schedule on non-RT days).	Duration: 6 months. Type: Supervised resistance training (RT), 3 days/week. Intensity: Progressive (40–85% 1-RM), 7 exercises, 2–3 sets, 5–15 reps.	Body mass (BM), fat mass (FM), fat-free mass (FFM), % body fat, BMI percentile, waist circumference (WC). Assessed via DXA.	No significant between-group differences in changes in BM, FM, FFM, % fat or WC. Significant within-group increases in BM, FM and FFM in all groups. FFM accounted for 62–74% of weight gain. Boys showed near-significant FM reduction in water vs. milk group (*p* = 0.054).	Conclusions: Milk supplementation during RT did not enhance body composition changes compared to isocaloric juice or water in overweight adolescents. Applications: Milk can be safely recommended alongside RT without adverse effects on body composition. Future studies should consider higher protein doses post-exercise and more frequent RT sessions (e.g., 5 days/week).
Minja et al. [4]	Sample: 745 children (396 girls, 349 boys) from 16 clusters at final follow-up (T3). Age: 6–12 years (Mean ~11.5 y at T3). Context: Rural primary schools in the Kilombero district, Tanzania. High prevalence of stunting (23–35%) and lower prevalence of overweight/obesity (8–13%).	Substance: Multi-micronutrient supplementation (MMNS) chewing tablet based on MixMe™ powder. Dosage: One tablet daily, 5 days/week.	Duration: 26 months (disrupted by COVID-19 school closures). Type: Two 45 min PA classes per week, including “moving to music” and structured “physical education” lessons based on the Kazikidz program. Intensity: Not specified in detail; lessons were designed to be playful and engaging.	Fat mass (FM), fat-free mass (FFM), truncal fat mass (TrFM), truncal fat-free mass (TrFFM) assessed via Bioelectrical Impedance Analysis (BIA).	Overall (primary outcome): No statistically significant differences in body composition were found across intervention groups compared to the control. Sex-stratified analysis: • Girls in PA group: Significant decrease in FM and FFM. • Girls in PA + MMNS group: Significant decrease in FFM and TrFFM. • Boys in MMNS group: Significant increase in FFM and TrFFM. • Boys in PA + MMNS group: Significant increase in FFM and TrFFM and a significant decrease in TrFM.	Conclusions: The interventions had no overall effect but revealed significant, contrasting gender-specific effects. MMNS promoted lean mass accumulation in boys, while PA (alone or combined) was associated with reductions in both fat and lean mass in girls. The combination of PA + MMNS was most effective for improving body composition in boys. Applications: School-based interventions combining PA and MMNS show potential as a “double-duty” strategy to address the dual burden of malnutrition. Programs must be tailored by gender to be effective, as boys and girls responded differently. Further research is needed to understand the causes of these sex differences and to design optimal interventions for both genders.
Nqweniso et al. [12]	Sample: 898 children (458 boys, 440 girls) from 8 schools. Age: 8–11 years. Context: Grade-4 children from quintile 3 (no-fee, government-funded) schools in Gqeberha (Port Elizabeth), South Africa. Schools were in low socioeconomic, historically disadvantaged townships.	Substance: Ready-to-Use Supplementary Food (RUSF), a peanut butter-based, energy-dense supplement. Dosage: One sachet (100 g, 530 kcal) administered once a day, 5 days a week for 10 weeks.	Duration: 10 weeks. Type: Varied by school. The PA intervention consisted of two 40 min structured PE lessons per week, one 40 min “moving-to-music” lesson, regular in-class activity breaks and adaptation of school playgrounds. Intensity: Not specified. Lessons were aligned with the prescribed PE curriculum and conducted by teachers assisted by external specialists.	Body Mass Index (BMI), BMI-for-age z-scores (BAZ), body fat percentage (BF%) measured via triceps and subscapular skinfold thickness using the Slaughter equation.	Overall: BMI, BAZ and BF% increased significantly from baseline to post-intervention in the total sample. By nutritional status: • Normal-weight children: The PA intervention (alone or combined with health education) mitigated increases in body fat levels compared to controls. • Overweight/Obese children: The PA intervention alone was associated with lower body fat increases compared to controls. • Nutrition intervention: The supplement (RUSF) did not show a significant beneficial effect on body composition; in one combined intervention group, the experimental group had a larger increase in BAZ than the control (though baseline was lower).	Conclusions: School-based physical activity interventions can be effective in mitigating unhealthy gains in body fat in both normal-weight and overweight/obese children from disadvantaged settings. The energy-dense nutritional supplement did not improve outcomes and may contribute to weight gain, highlighting that supplementation must be appropriately targeted. Applications: PA should be promoted within schools to combat rising obesity. Interventions must be tailored to the population’s needs; energy supplementation is not suitable for general school populations and should be reserved for undernourished sub-groups. The study underscores the challenge of the “double burden of malnutrition” and the need for precise public health strategies.
Long et al. [13]	Sample: 1227 children (586 girls, 641 boys) from original 1304 Age: 6–12 years (Mean ~8.3 y) Context: Peri-urban, disadvantaged communities (Quintile 3 schools) in Gqeberha, South Africa. High prevalence of stunting (~9%) and overweight/obesity (~15%).	Substance: Multi-micronutrient supplementation (MMNS) chewing tablet based on MixMe™ powder sprinkle. Modified by replacing vitamin A with 4500 µg β-carotene. Dosage: One tablet daily, 5 days/week.	Type: 1. Daily in-class activity breaks. 2. Weekly 45–60 min playful physical education lessons. 3. Weekly 45–60 min dancing-to-music lessons. Intensity: Not specified in detail; lessons were designed to be playful and engaging.	Fat mass (FM), fat-free mass (FFM), truncal fat mass (TrFM), truncal fat-free mass (TrFFM) assessed via Bioelectrical Impedance Analysis (BIA).	At 9 months (T2): • PA arm was associated with reduced FM (*p* = 0.03) and reduced TrFM (*p* < 0.01) in the full cohort. • MMNS arm was associated with increased FFM (*p* < 0.01). • Sex-stratified: Significant effects were found predominantly among girls (PA reduced FM and TrFM; MMNS increased FFM). Effects among boys were minimal. • Growth-stratified: Children with lower height velocity in the PA and MMNS arms showed greater improvements (reduced FM/TrFM, increased FFM) compared to controls. • No significant synergistic effect was found for the combined PA + MMNS arm.	Conclusions: School-based physical activity promotion reduces fat mass, while multi-micronutrient supplementation increases fat-free mass. These effects are most pronounced in girls and in children with poorer growth patterns. The interventions work through distinct pathways. Applications: Incorporating PA promotion and MMNS into school health programs represents an effective “double-duty” strategy to simultaneously address the dual burden of malnutrition (undernutrition and obesity) in disadvantaged settings, particularly for vulnerable subgroups like girls and growth-impaired children.
Long et al. [14]	Sample: 1304 children (637 girls, 667 boys) Age: 6–12 years Context: Peri-urban, disadvantaged communities (Quintile 3 schools) in Gqeberha, South Africa	Substance: Multi-micronutrient supplementation (MMNS) tablet based on MixMe™ powder sprinkle. Modified to replace Vitamin A with 4500 µg β-carotene. Contains iron, zinc, etc. Dosage: One tablet daily, 5 days/week.	Duration: 21 months (intervention disrupted by COVID-19 school closures) Type: 1. Daily in-class activity breaks. 2. Weekly 45–60 min structured physical education lessons. 3. Weekly 45–60 min dancing-to-music lessons. Intensity: Not specified in detail; lessons were designed to be playful and engaging.	Fat-free mass (FFM), truncal FFM, fat mass (FM), truncal FM (via BIA). Micronutrient status (Vitamin D, Zinc, RBP for Vit A, sTfR for iron).	At 21 months (T3): • Direct intervention effects were not sustained, likely due to COVID-19 disruptions. • Zinc-mediated indirect effects on FFM remained significant for both periods. • No significant associations found for Vitamin D or RBP with body composition changes.	Applications: Combining PA and MMNS in school-based programs is a promising strategy to improve body composition and micronutrient status in children from disadvantaged settings. Program sustainability and resilience to external disruptions are key to long-term success.
Doaei et al. [18]	Sample: 90 male overweight/obese adolescents Age: 12–16 years (mean ≈13.9 y) Context: Urban, Tehran, Iran	Substance: None (grouped by baseline serum 25(OH)D; no exogenous vitamin D supplement administered) Dosage: N/A	Duration: 12 weeks Type: Comprehensive school-based lifestyle intervention. Physical component: High-Intensity Interval Training (HIIT). Intensity: High-intensity. Sessions: 10 min warm-up + ≥30 min high-intensity exercise.	Height, mass, BMI, body fat %, skeletal muscle %, serum 25(OH)D.	Baseline: Obese participants had significantly lower serum 25(OH)D than overweight participants (37.67 vs. 44.01 ng/mL, *p* < 0.04). The high vitamin D group (≥40 ng/mL) had significantly lower body weight (*p* < 0.01). Post-18 weeks: No significant correlations were found between the changes in vitamin D levels (Δ25(OH)D) and the changes in any anthropometric measurements (Δweight, ΔBMI, Δfat%, Δmuscle%).	Conclusions: Serum vitamin D level is inversely associated with obesity status but is not associated with short-term changes in body weight or composition from an 18-week lifestyle intervention in male adolescents. Applications: The study highlights that vitamin D status may be a marker of obesity but not a modifiable target for short-term weight loss interventions in this demographic. Future research should focus on underlying mediating factors.

Note. DXA = dual-energy X-ray absorptiometry, PA = physical activity, PE = physical education, RBP = retinol binding protein, Vit A = vitamin A, sTfR = serum transferrin receptor, 25(OH)D = primary circulating form of vitamin D in the body.

## Data Availability

The data presented in this study are available within the article.
